# Signal Transduction of Sphingosine-1-Phosphate G Protein—Coupled Receptors

**DOI:** 10.1100/tsw.2006.182

**Published:** 2006-08-11

**Authors:** Nicholas Young, James R. Van Brocklyn

**Affiliations:** ^1^ Integrated Biomedical Science Graduate Program, Ohio State University, Columbus, USA; ^2^ Department of Pathology, Ohio State University, Columbus, USA

**Keywords:** sphingosine-1-phosphate, S1P, EDG, cell proliferation, cell migration, angiogenesis, lymphocyte trafficking, cancer

## Abstract

Sphingosine-1-phosphate (S1P) is a bioactive lipid capable of eliciting dramatic effects in a variety of cell types. Signaling by this molecule is by a family of five G protein—coupled receptors named S1P_1–5_ that signal through a variety of pathways to regulate cell proliferation, migration, cytoskeletal organization, and differentiation. These receptors are expressed in a wide variety of tissues and cell types, and their cellular effects contribute to important biological and pathological functions of S1P in many processes, including angiogenesis, vascular development, lymphocyte trafficking, and cancer. This review will focus on the current progress in the field of S1P receptor signaling and biology.

## INTRODUCTION

The bioactive lipid sphingosine-1-phosphate (S1P) is an agonist for a subset of G protein–coupled receptors (GPCRs) known as the endothelial differentiation gene (EDG) family. S1P is present at high concentrations in the blood[1], roughly 0.1–1 μ*M*, where it is bound to albumin and to lipoproteins[2]. It is secreted by platelets on activation[3] and by mast cells[4], as well as by a variety of other cell types. It is generally thought to be ubiquitously present at low levels outside of the bloodstream within tissues.

S1P is formed by phosphorylation of sphingosine, the long-chain base that serves as the backbone of sphingolipids, by the ubiquitously expressed enzyme sphingosine kinase (SphK). SphK displays basal activity in unstimulated cells, however, it also can be activated by a variety of signals, including growth factors[5,6,7,8], immunoglobulin receptors[9,10], tumor necrosis factor[11], and various GPCRs[12,13]. As SphK is a cytoplasmic-signaling enzyme, it was originally thought that S1P functioned within cells as a second messenger. Interestingly, although S1P is now known to function at the cell surface by activating several GPCRs, significant evidence exists that S1P also has receptor-independent, intracellular functions[14], although definitive proof of this awaits the identification of a direct intracellular target.

Since the discovery of the S1P family of receptors in the late 1990s, the modulation of cellular and biological functions by S1P receptor stimulation has been under intense investigation. Five related GPCRs that specifically bind S1P with high affinity have been identified, including S1P$_1$/EDG-1/LP$_{B1}$, S1P$_2$/EDG-5/H218/AGR16/LP$_{B2}$, S1P$_3$/EDG-3/LP$_{B3}$, S1P$_4$/EDG-6/LP$_{B4}$, and S1P$_5$/EDG-8/NRG-1. Although a variety of naming systems for these receptors have been used, the currently accepted system, based on International Union of Pharmacology (IUPHAR) guidelines, is the S1P nomenclature[15] and, therefore, that system will be used in this review, regardless of which names were used in the original papers. Each of the five S1P receptors has significant homology displaying 40–50% amino acid identity and approximately 60% amino acid similarity[16,17,18]. Several other orphan receptors have been implicated as potential S1P receptors, including GPR3, 6, 12, and 63, although these remain unconfirmed and little is known regarding the significance of these receptors (reviewed in [19]).

$S1P_1$, $S1P_2$, and $S1P_3$ were originally orphan receptors. The $S1P_1$ receptor subtype was the first of the family to be cloned and was identified as an immediate early transcript in phorbol ester–induced endothelial cell differentiation[20], leading to the designation endothelial differentiation gene (EDG)-1. $S1P_2$ was cloned from rat brain and rat vascular smooth muscle cells(VSMC)[21,22], while $S1P_3$ was cloned from a human genomic library[23]. The identification of a related receptor, EDG-2/vzg-1, as a receptor for lysophosphatidic acid (LPA)[24], another bloodborne lipid that is structurally similar to S1P and has several overlapping cellular effects, suggested that S1P may be a ligand for other EDG family receptors. This led to the identification of $S1P_1$ as a specific S1P receptor[25]. Shortly after the identification of EDG-2/$LPA_1$ as the first LPA receptor, $S1P_2$ and $S1P_3$ were shown to mediate S1P-induced transcription from a serum response, element-driven promoter[26]. $S1P_4$ was later cloned from *in vitro* differentiated murine and human dendritic cells by a PCR-based strategy using degenerate primers based on other EDG receptors[27]. $S1P_5$ was identified as a nerve growth factor down-regulated gene based on EST profiling and cloned from a rat PC12 cell cDNA library[28].

S1P regulates a wide range of cellular effects and biological responses. S1P stimulates proliferation of several cell types, although it can inhibit proliferation in some cases. S1P affects cell migration, again both positively and negatively depending on the receptors expressed. S1P can also block apoptosis induced by a variety of stimuli. S1P can affect cellular differentiation and is important in some aspects of development. S1P has also recently been shown to be a critical regulator of immune cell trafficking and an important regulator of tumor angiogenesis. These responses have been linked to the various GPCRs that S1P regulates and will be discussed in detail below.

Each S1P receptor subtype signals through a unique set of G proteins that result in various downstream cellular effects. Therefore, it is critical to know what receptors are expressed in order to predict cellular behavior on S1P stimulation. Of the four major families of heterotrimeric G proteins, $G_s$, $G_{i/o}$, $G_{q/11}$, and $G_{12/13}$ S1P receptors couple to $G_i$, $G_q$, and $G_{12/13}$[29]. Thus, S1P leads to activation of the classical pathways downstream of these G proteins, adenylyl cyclase inhibition as well as the Ras/MAP kinase cascade and phosphatidylinositol 3-phosphate kinase (PI3 kinase)/Akt for $G_i$, phospholipase C (PLC) for $G_q$, and small Rho GTPases for $G_{12/13}$. The particular pathways activated by various receptors and the biology that they regulate will be discussed.

## LIGAND AFFINITIES AND SPECIFICITIES OF S1P RECEPTORS

All five S1P receptors bind S1P with nanomolar affinities although precise values vary somewhat between experiments. $S1P_1$ has been shown to bind S1P with $K_D$s ranging from 8–50 n*M*[25,30,31,32,33]. The mean value from the affinity measurements for $S1P_1$ reported in these papers is 24.7 ± 17.8 n*M*. Similar values are obtained for $S1P_2$ (18.4 ± 7.2 n*M*) and $S1P_3$ (19.7 ± 8.6 n*M*)[25,30,31,32,33,34]. $S1P_5$ appears to bind S1P with slightly higher affinity, as this has been reported to be 2[17] and 6 n*M*[35]. Reports of $S1P_4$ binding affinity for S1P have varied more widely, including 12.8[18], 63[36], 95, and even 156 n*M*[37]. Notably, all of these studies except the Yamazaki et al. paper found that $S1P_4$ has the lowest binding affinity for S1P of the five S1P receptors. Interestingly, $S1P_4$ has a much higher affinity (approximately 2 n*M*) for phytosphingosine-1-phosphate (phS1P), which has a 4-hydroxyl group and is commonly found in plants[37]. As this is a minor lipid in mammalian cells, the biological significance of this finding is uncertain. Interestingly, one study showed significantly higher-affinity binding of S1P to membranes from $S1P_1$ $S1P_2$, $S1P_3$, and $S1P_5$ overexpressing cells, all ranging from 0.17–0.61 n*M*[38].

The reason for this discrepancy is unclear. Nevertheless, as concentrations of S1P in plasma and serum range from approximately 100 n*M* to 1 μ*M*[1,39], it is apparent that all the above numbers represent physiologically relevant affinities.

Several other sphingolipids, related in structure to S1P, have been tested as potential ligands for S1P receptors. The majority of these lipids have shown no binding or agonist properties for these receptors. The exceptions are dihydrosphingosine-1-phosphate (dhS1P), which lacks the trans-double bond at the 4 position, and sphingosylphosphorylcholine (SPC), which is similar to S1P, but contains a choline head group, i.e., lysosphingomyelin. dhS1P is an agonist for all five S1P receptors. For $S1P_1$ and $S1P_3$, dhS1P binds and activates the receptors with a similar affinity to S1P[33]. Interestingly, at $S1P_2$, dhS1P is less potent, competing for S1P binding with an $IC_{50}$ of approximately 200 n*M*[33]. One study showed an $IC_{50}$ for dhS1P for $S1P_4$ of approximately 9 n*M*[37], significantly higher than its affinity for S1P. Similar numbers were obtained in another study that also showed that dhS1P bound $S1P_4$ with an affinity similar to that of phS1P, approximately 4 n*M*, while S1P bound with 10- to 20-fold lower affinity[40]. SPC also functions as an agonist for the five S1P receptors, however, for all five, it is a low-affinity agonist with $IC_{50}$s for binding and $EC_{50}$s for various signaling responses generally occurring in the low micromolar range[17,30,36,41,42,43].

## G PROTEIN COUPLING OF S1P RECEPTORS AND PATHWAYS ACTIVATED

Prior to the identification of S1P as its ligand, $S1P_1$ was shown to couple to $G_i$ and the extracellular signal regulated kinase (ERK) mitogen-activated protein (MAP) kinase pathway[44]. GTPγS binding studies showed that $S1P_1$ couples exclusively to $G_i$ proteins[29] and, thus, responses mediated through this receptor are sensitive to pertussis toxin (PTX), which ADP-ribosylates and inactivates $G_{i/o}$ proteins. $S1P_1$ activation by S1P has been shown to lead to the activation of several signal transduction pathways (including ERK[25,45]), inhibition of forskolin-stimulated cAMP accumulation[46], and increased $[Ca^{2+}]_i$ in CHO cells[30], although the calcium response was not detected in HEK293 cells overexpressing $S1P_1$[46] and in *Xenopus* oocytes, $S1P_1$ required coexpression of a chimeric $G\alpha_{q/i}$ protein, allowing $S1P_1$ to couple to phospholipase C in order to affect $[Ca^{2+}]_i$[43]. The increased $[Ca^{2+}]_i$ seen with $S1P_1$ signaling in some cell types may be mediated by $G_i$-induced activation of SphK, leading to intracellular production of S1P- and $IP_3$-independent calcium release from intracellular stores[47]. On the other hand, $S1P_1$ signaling can inhibit calcium responses induced by various other signals[48].

$S1P_1$ activates PI3 kinase, leading to Akt activation and endothelial nitric oxide synthase (eNOS) phosphorylation in endothelial cells[49]. The activation of this pathway may be affected by the subcellular localization of $S1P_1$. In this regard, S1P stimulation of $S1P_1$ causes its accumulation in caveolae[50]. Interaction of $S1P_1$ with caveolin leads to a decrease in its ability to activate eNOS[50] as well as Akt and the small GTPase Rac. Interestingly, caveolin interaction does not affect $S1P_1$-induced ERK activation[51]. Caveolar localization of $S1P_1$ requires N-glycosylation, as does receptor internalization[52].

Several studies have also shown interesting cooperative interactions of $S1P_1$ and platelet-derived growth factor (PDGF) signaling. Spiegel's group demonstrated that $S1P_1$ expression was necessary for PDGF-induced Rac activation and chemotaxis in HEK293 cells and mouse embryonic fibroblasts (MEFs)[53]. Since PDGF activates SphK, and led to $S1P_1$ phosphorylation, they suggested an autocrine model in which PDGF induces production of S1P that in turn activates $S1P_1$ leading to Rac activation and cell migration. The same group also showed that PDGF required $S1P_1$ to induce membrane ruffles and that PDGF causes SphK to translocate to membrane ruffles, suggesting that local S1P formation at the leading edge might be involved in directed cell migration[54]. $S1P_1$ was also involved in PDGF-induced FAK, src, and p38 activation[54]. In addition, Pyne's group has shown that the PDGF receptor and $S1P_1$ are physically linked. In their "integrative" model, the PDGF receptor phosphorylates $G_{\alpha i}$, which is recruited by $S1P_1$, and this step enhances signaling by both $S1P_1$ and the PDGF receptor[55,56]. The same group further found that PDGF-induced cell migration was blocked by an inverse agonist of

$S1P_1$[57]. It is unclear which of the above models is more correct, nor are the two ideas are necessarily mutually exclusive. However, it is possible that this system may function differently in different cell types. In this regard, $S1P_1$ was found not to be required for PDGF-induced chemotaxis of rat VSMC and MEFs from $S1P_1$ knockout embryos[58]. To further add to the complexity of the S1P/PDGF interplay, $S1P_1$ signaling was recently found to induce expression of PDGF-A and PDGF-B[59], and S1P has been shown to transactivate epidermal growth factor receptor (EGFR) and PDGFR in VSMC[60] although the S1P receptor involved was not identified.

$S1P_2$ and $S1P_3$ both couple to $G_i$, $G_q$, and $G_{13}$[29], however, it appears that they do so with different preferences, leading to different biological responses. As both of these receptors link to $G_i$ activation, they activate ERK MAP kinase in a PTX-sensitive fashion, as does $S1P_1$. Interestingly, while the potency of ERK activation by $S1P_3$ is similar to that of $S1P_1$, it is higher than that mediated by $S1P_2$[41]. Both are also capable of activating PLC to induce $IP_3$ formation and $Ca^{2+}$ mobilization in a partially PTX-sensitive fashion[31,41], suggesting involvement of both $G_i$ and $G_q$. However, $S1P_3$ more potently stimulates inositol phosphate production and increased $[Ca^{2+}]_i$[31]. On the other hand, $S1P_2$ couples strongly to activation of the small GTPase Rho and stress fiber formation[42]. $S1P_2$ also is a potent inducer of cell rounding and process retraction in PC12 cells[34], a process linked to Rho activation[61]. Results using cells from $S1P_2$ and $S1P_3$ knockout mice support this distinction. Analysis of S1P signaling in MEFs from $S1P_2^{-/-}$ embryos revealed a defect in Rho activation, but no change in calcium mobilization, adenylyl cyclase inhibition, or inositol phosphate production[62]. In contrast, MEFs from $S1P_3^{-/-}$ embryos displayed a loss of PLC activation and calcium mobilization, but with only a minor effect on Rho activation[62]. Thus, it appears that $S1P_2$ couples preferentially to $G_{12/13}$, leading to potent Rho activation, while $S1P_3$ prefers $G_q$, thus favoring PLC activation. Nevertheless, $S1P_2$ couples to $G_{\alpha q}$, $G_{\alpha 13}$, and $G_{\alpha i2}$ in smooth muscle cells and leads to PLC as well as Rho activation[63]. Thus, the extent of coupling to various G protein and signaling pathways activated by the various receptors will likely vary in different cellular contexts.

$S1P_2$ has also been shown to activate p38 and Jun N-terminal kinase (JNK) MAP kinases[42] and phospholipase D activation[32], all by PTX-insensitive mechanisms. Both $S1P_2$ and $S1P_3$ also activate NF-κB, probably via $G_q$[64]. As expected for $G_i$-coupled receptors, $S1P_2$ and $S1P_3$ inhibit forskolin-induced cAMP accumulation[31]. Interestingly, however, in the absence of forskolin, signaling by these receptors leads to increased cAMP[31]. $S1P_1$ causes this effect as well, although it is much weaker in this capacity.

Recently, $S1P_3$ has also been shown to participate in cross-talk with a receptor tyrosine kinase. $S1P_3$ causes PDGFR transactivation, leading to Akt activation[65]. $S1P_3$ also causes transactivation of EGFR via a Src family kinase and matrix metalloproteinase-dependent release of heparin-binding EGF-like growth factor (HB-EGF)[66]. Interestingly, via this pathway, $S1P_3$ mediates EGFR transactivation by estrogen receptors that activate SphK, leading to S1P secretion[66].

$S1P_4$ couples to both $G_i$ and $G_{12/13}$, but not $G_q$[67]. This specific G protein coupling has distinct downstream signaling effects that influence a variety of cellular activities. $S1P_4$ can lead to activation of PLC, ERK, and the Rho family small GTPase Cdc42 through $G_i$ coupling[18,36,68]. Cdc42 activation by $S1P_4$ positively regulates cellular motility[68]. Coupling of $S1P_4$ to $G_{12/13}$ leads to Rho activation, formation of peripheral stress fibers, and cell rounding[67].

$S1P_5$ couples to both $G_i$ and $G_{12}$, but not $G_q$[35]. $G_i$ signaling leads to inhibition of cAMP accumulation in both CHO and RH7777 cells in a PTX-sensitive manner[17,35]. Surprisingly, however, $S1P_5$ is unusual compared to other S1P receptors as it causes a decrease in ERK1/2 phosphorylation and activity[35]. Repression of ERK by $S1P_5$ is PTX insensitive, but is prevented by the phosphatase inhibitors orthovanadate and okadaic acid, indicating a role for a phosphatase[35]. Furthermore, $S1P_5$ activates JNK MAP kinase, but not p38[35].

## REGULATION OF CELL PROLIFERATION AND SURVIVAL

The first biological effect defined for S1P, in the early 1990s, was mitogenesis[69]. PTX sensitivity of the mitogenic effect of S1P on fibroblasts suggested that GPCRs were involved in this response[70]. Nevertheless, it was, and still is, unclear to what degree receptors vs. intracellular receptor–independent effects of S1P contribute to mitogenesis. However, significant evidence exists that S1P receptors do, in some cases, stimulate cell proliferation.

S1P has been shown to stimulate proliferation of a variety of cell types, including fibroblasts[69], VSMC[71], osteoblasts[72], breast cancer cells[73], hepatic stellate cells[74], endothelial cells[75], airway smooth muscle[76], astrocytes[77], mesangial cells[78], intestinal epithelial cells[79], glioma cells[80], neural progenitor cells[81], and chondrocytes[82].

Multiple S1P receptors and signaling pathways can contribute to the mitogenic effect of S1P. S1P-induced proliferation of VSMC was augmented by overexpression of $S1P_1$ through activation of p70 S6 kinase and cyclin D1 expression[71]. S1P stimulated proliferation of glioma cells that express $S1P_1$, $S1P_2$, and $S1P_3$ through ERK and PI3-kinase $\beta$ activation[80]. S1P-induced proliferation of breast cancer cells, expressing $S1P_2$ and $S1P_3$, but not $S1P_1$, was mediated by direct activation of serum-response element signaling and by induction of IGF-II expression[73]. Furthermore, overexpression of $S1P_2$ or $S1P_3$ in hepatoma cells allowed S1P to stimulate proliferation[83].

S1P can also exert antiproliferative effects in several cell types. S1P has an antiproliferative effect in hepatocytes[84]. S1P also blocks insulin and TSH-stimulated proliferation of thyroid cells[85]. In hepatic myofibroblasts, S1P blocks proliferation through induction of cyclooxygenase-2 (COX-2) and production of prostaglandin $E_2$ and cAMP[86]. Although the receptor(s) involved were not identified, this effect was PTX insensitive. S1P also suppresses proliferation of T lymphocytes[87,88], keratinocytes[89], and gastric cancer cells[90].

$S1P_2$ may be a major regulator of the antiproliferative effects of S1P, as $S1P_2^{-/-}$ MEFs proliferate more rapidly than wild type MEFs[91]. Restoration of $S1P_2$ expression decreases proliferation[91]. Furthermore, S1P inhibition of hepatocyte proliferation was mediated by $S1P_2$-induced Rho activation[84]. On the other hand, suppression of T cell proliferation by S1P is mediated by $S1P_1$[87]. In addition, $S1P_5$ overexpression inhibits proliferation of CHO cells[35]. Thus, as with positive proliferation responses, antiproliferative responses to S1P can be mediated by various receptors. Moreover, in some cases, the same S1P receptors can stimulate proliferation in some cell types, while inhibiting it in others.

S1P inhibits apoptosis induced by a variety of signals and insults[13]. Inhibition of apoptosis by S1P may also be mediated by several pathways, however, the PI3 kinase/Akt pathway appears to be particularly important. As $S1P_1$ has most commonly been linked with this pathway, this may be the primary receptor involved in the survival response, although $S1P_2$ and $S1P_3$ have also been implicated in some cases. For example, S1P-induced Akt activation promotes survival of ovarian cancer cells[92]. Protection of endothelial cells from apoptosis by S1P is mediated by $S1P_1$ and $S1P_3$ signaling through Akt to activate eNOS[93]. Although S1P blocks proliferation of melanocytes, it enhances cell survival through activation of ERK and Akt[94]. Interestingly, S1P can activate both pro- and antiapoptotic effects in the same cell type. In this regard, it was found that S1P stimulates apoptosis of hepatic myofibroblasts by a receptor-independent mechanism, while activating PI3 kinase to inhibit apoptotic signaling[95]. S1P also enhances neutrophil survival through the p38 pathway[96]. In addition, protection of T lymphoblastoma cells from apoptosis by S1P appears to involve $S1P_2$ and $S1P_3$ signaling and decreased expression of the proapoptotic protein Bax[97].

## REGULATION OF CELL MIGRATION

A great deal of work has been done in the analysis of S1P signaling in the cellular migratory response. Early studies showed that S1P inhibited migration of several cell types including melanoma, fibrosarcoma[98], and breast cancer cells[99]. In contrast, S1P potently stimulates motility of endothelial cells[100,101,102], some glioma cells[103] and, at low concentrations, T cells[104]. In some cases, S1P enhances VSMC migration[105,106] while inhibiting movement of these cells in others[107].

The differential influence of S1P on cell migration is dependent on the receptor subtypes expressed by the cell. Overexpression of $S1P_1$ or $S1P_3$ in CHO cells allowed for S1P-induced cell migration, while $S1P_2$ overexpression had no effect[31]. Moreover, $S1P_2$ overexpression caused inhibition of Rac activation, membrane ruffling, and cell migration[108]. Furthermore, $S1P_2$ overexpression decreases stimulation of endothelial cell migration, while $S1P_1$ overexpression in smooth muscle cells reduces inhibition of migration[107] or, in some cases, allows S1P-stimulation of migration[71]. The inhibitory regulation of Rac by $S1P_2$ was shown to be downstream of Rho, which is potently activated by $S1P_2$ through $G_{12/13}$[109]. Inhibition of $G_i$ signaling by $S1P_3$ using PTX allowed S1P to inhibit migration through this receptor as well. Thus, the overall picture is that $S1P_2$ preferentially couples to $G_{12/13}$ to inhibit Rac and cell migration, while only weekly activating $G_i$, and $S1P_3$ activates $G_i$ and $G_q$ more potently, thus stimulating migration. $S1P_1$, coupling only to $G_i$, enhances cell migration.

In contrast, a more recent study has shown that S1P stimulation of $S1P_2$ in glioma cells actually increases Rac activation and that the decrease in observed migration in these cells is due to Rho signaling through Rho-associated protein kinase (ROCK), as ROCK inhibition reversed the effect[110]. Additionally, this study showed stress fiber formation by $S1P_2$ signaling and cell rounding on overexpression of $S1P_2$. Thus, the $S1P_2$ receptor consistently opposes migration, but does so through different signaling mechanisms, i.e., inhibition of Rac in some cell types and ROCK-mediated stress fiber formation in others. Through Rho signaling, $S1P_2$ can also activate PTEN, a phosphatase acting on 3-phosphorylated phosphoinositides and thus counteracting PI3 kinase signaling, to inhibit cell migration[111].

$S1P_4$ may also be able to stimulate cell migration. Overexpression of $S1P_4$ allowed S1P to stimulate migration of CHO cells through activation of another small GTPase, Cdc42[68]. Also, mutation of a zebrafish S1P receptor Miles Apart (Mil) prevented migration of heart precursors to the midline[112]. Interestingly, Mil expression was not required in the heart precursors themselves, suggesting that Mil established a permissive environment for heart precursor migration.

## S1P RECEPTOR EXPRESSION

$S1P_1$, $S1P_2$, and $S1P_3$ are widely expressed in most tissues at various levels[16], suggesting that these receptors influence many cellular processes. $S1P_1$ is expressed at high levels in brain, heart, lung, liver, and spleen; moderate levels in kidney, muscle, and thymus; and at low levels in several other tissues[16,113]. On a cellular level, $S1P_1$ is expressed in endothelial cells of most tissues, although not in spleen, kidney, and testis[113]. Expression was also detected in pulmonary smooth muscle, cardiomyocytes, hepatocytes, neurons, especially Purkinje cells, astrocytes, spermatids, spleen marginal zone, and the collecting ducts of the kidney[113]. Mouse splenic T cells also express $S1P_1$[104]. During embryogenesis, $S1P_1$ was induced late in development (after embryonic day 15.5) at centers of ossification[114]. $S1P_1$ is also expressed in areas of neurogenesis adjacent to ventricles and in areas of angiogenesis in developing brain[115].

$S1P_2$ is also ubiquitously expressed in adult mice, but is present at the highest levels in heart and lung. In embryonic rat, $S1P_2$ was expressed in lung, kidney, skin, gut, spleen, brain, and liver[22], however, in E14–18 mouse embryos, $S1P_2$ was only detected in the brain[16]. Brain expression decreased significantly postnataly[22]. $S1P_2$ is expressed in young neuronal cell bodies and later is found in growing axons[116]. In addition, NGF treatment of PC12 cells led to decreased $S1P_2$ expression[22].

$S1P_3$ is expressed at high levels in adult mouse heart, lung, kidney, and spleen with lower levels seen in most other tissues[16]. Northern analysis of human RNA confirmed high $S1P_3$ expression in heart and kidney, but showed lower expression in lung[23]. Expression was also high in human liver, pancreas, and placenta[23], while liver expression was low in the mouse[16].

In contrast to $S1P_{1–3}$, $S1P_4$ and $S1P_5$ are more restricted in their expression. $S1P_4$ expression is limited principally to lymphoid tissue and hematopoietic cells, and lung in both adults and embryos[27]. With the exception of $S1P_4$, all S1P receptor subtypes have been observed to be expressed in the central nervous system (CNS). This appears to be the predominant location of $S1P_5$ expression. Northern analysis further revealed expression of $S1P_5$ prominently throughout the rat brain[17,28]. More specifically, this receptor subtype was strongly detected by *in situ* hybridization in the white matter tracts throughout the CNS[17]. In postnatal day 21, mouse brain $S1P_5$ expression was detected exclusively in oligodendrocyte precursors and mature oligodendrocytes, although in the mature cells $S1P_5$ was associated with the myelin sheath and not the cell body[117]. Radial glial cells in human fetal brain also express $S1P_5$[118]. In addition, $S1P_5$ expression has also been detected in the spleen[17] and skin[119].

Clearly, many tissues and individual cell types coexpress several S1P receptor subtypes. In addition, as noted above, the various receptors affect overlapping, but not identical, sets of signaling pathways. As a result, it is likely that many of the biological responses to S1P are mediated by the coordinated effects of multiple S1P receptor subtypes. In addition, S1P receptors heterodimerize among the various receptor subtypes[120], suggesting possible functional interactive effects of coexpressed receptors.

## BIOLOGICAL ROLES OF S1P RECEPTORS DETERMINED FROM KNOCKOUT MICE

Results from $S1P_1$ knockout mice revealed an important role for this receptor in embryonic development. $S1P_1^{-/-}$ embryos died between E12.5 and E14.5[121]. The embryonic lethality was attributed to massive hemorrhage due to failure of VSMC to cover the dorsal surface of blood vessels. Although this highlights a clear role for $S1P_1$ in vascular maturation, the endothelium formed a normal vascular network as shown by immunohistochemical staining for endothelial cell markers. Therefore, normal vasculogenesis and angiogenesis apparently occurred. Nevertheless, a conditional mutant, in which $S1P_1$ was knocked out only in endothelial cells, displayed the same phenotype, indicating that although the defect lies in smooth muscle migration to cover blood vessels, $S1P_1$ expression is required in the endothelial cells[122]. $S1P_1$ expression in endothelial cells is also necessary for proper limb development and digit formation[123]. Thus $S1P_1$ expression in endothelial cells may be important for creating a permissive environment for several developmental processes. Furthermore, $S1P^{-/-}$ embryos displayed a significant loss of cells in the developing brain due to increased apoptosis and decreased mitosis, indicating an important role for $S1P_1$ in neurogenesis[124].

$S1P_2$ knockout mice have been reported from two independent investigations. The $S1P_2^{-/-}$ mice had a much milder phenotype than the $S1P_1$ knockout in both instances, being born apparently normal, which suggests a possible redundant signaling role with other S1P receptors. Modest decreases in litter size were observed in one study in otherwise normal mice[62]. In contrast, another group detected seizures due to increased excitability of neocortical pyramidal neurons, which were occasionally fatal[125]. These receptor null mice were normal in all other phenotypic areas investigated.

$S1P_3$ receptor knockout mice are also born apparently normal[119]. The litter sizes from homozygous $S1P_3^{-/-}$ mice crosses were slightly smaller than normal. $S1P_3$ deletion led to a compensatory increase in expression of $S1P_2$ in the heart and brain[119].

The double knockout mouse for $S1P_2$ and $S1P_3$ showed a dramatically reduced litter size and marked perinatal lethality, although surviving double knockout mice appeared normal[62]. The reduced litter size in $S1P_2$/$S1P_3$ double knockout mice appears to be the result of similar vascular defects in many embryos due to endothelial cells with abnormally thin cell bodies leading to bleeding[126]. On a cellular level, double knockout MEFs displayed a complete lack of Rho activation and severely reduced PLC activation with no loss of adenylyl cyclase inhibition[62]. $S1P_1$/$S1P_2$ double knockouts show a more severe phenotype than $S1P_1$ knockout alone, dying approximately 2 days sooner, with $S1P_1$/$S1P_2$/$S1P_3$ triple knockouts slightly more severe[126]. This was due to a less-well-formed vasculature containing fewer capillary branches in the head. Thus, it seems that $S1P_1$, $S1P_2$ and $S1P_3$ cooperate in the process of vascular formation.

$S1P_4$ knockout mice have not yet been reported, but an $S1P_5$ receptor knockout mouse has been reported[117]. Although $S1P_5$ expression was found in both oligodendrocyte precursors and mature oligodendrocytes, myelination in brains of $S1P_5^{-/-}$ mice appears normal[117]. $S1P_5$ was shown to influence both survival and process retraction of oligodendrocytes depending on the status of cellular differentiation[117]. Preoligodendrocytes stimulated with S1P resulted in process retraction, while the mature cells activated Akt to promote survival in a PTX-sensitive manner.

## ANGIOGENESIS

As noted above, the cloning of $S1P_1$ from differentiating endothelial cells suggested a potential role for this receptor in angiogenesis. Indeed, one of the first biological responses that was demonstrated downstream of $S1P_1$ signaling was induction of morphogenetic differentiation of endothelial cells, i.e., the formation of networks resembling vascular tubes[25]. S1P induction of endothelial morphogenetic differentiation requires $S1P_1$-dependent enhancement of cell survival, $S1P_1$ and $S1P_3$ regulated cytoskeletal changes, and adherens junction assembly[127]. Enhanced endothelial cell survival is mediated by activation of eNOS[93], which is activated through PI3-kinase β[128], whereas cytoskeletal responses required $S1P_1$-induced Rac activation and $S1P_3$-induced Rho activation, leading to adherens junction assembly[127].

S1P is also potently chemotactic for endothelial cells[100,101,102], and this response requires $S1P_1$ and $S1P_3$ signaling[129,130], leading to Rho-dependent clustering of integrins at focal adhesions and Rac activation[130]. Inhibition of Rho or p38 MAP kinase blocks S1P-induced endothelial cell migration[131]. Furthermore, $S1P_1$-induced endothelial cell migration requires phosphorylation of the $S1P_1$ receptor itself by Akt, and this step is required for Rac activation and angiogenesis[132]. This Akt-mediated $S1P_1$ phosphorylation/Rac pathway, regulates the formation of a cortactin-actin–related protein (Arp)2/3 complex, which ultimately results in membrane ruffling, formation of lamellipodia, and endothelial migration[133]. These profound effects of S1P signaling on endothelial cells lead to potent stimulation of angiogenesis. S1P stimulates angiogenesis *in vivo* in the matrigel plug assay[134], and synergizes with FGF-2 and VEGF to induce *in vivo* angiogenesis by an $S1P_1$- and $S1P_3$-dependent mechanism[102,127]. Synergism with VEGF may be related to the induction of $S1P_1$ expression by VEGF[135]. Moreover, S1P transactivates VEGFR in endothelial cells and the VEGFR-2/flt-1/KDR was required for S1P-induced Akt and eNOS activation[136], and membrane ruffling and motility[137].

The importance of $S1P_1$ for endothelial cell function was emphasized by one study showing that siRNA-mediated $S1P_1$ knock down eliminated S1P induction of actin ruffles and angiogenesis, sensitized cells to oxidant-mediated injury, and decreased expression of several adhesion molecules[138]. $S1P_3$ also appears to be important in the angiogenic response to S1P, as a peptide design to mimic the second intracellular loop of $S1P_3$, specifically caused $S1P_3$ internalization, and mimicked the effects of S1P on stimulating angiogenesis[139]. In addition, $S1P_1$ and $S1P_3$ mediate HDL-induced stimulation of endothelial cell migration, while $S1P_1$ mediates HDL-induced endothelial cell survival[140]. $S1P_2$, on the other hand, appears to antagonize the proangiogenic effects of $S1P_1$ and $S1P_3$, as inhibition of $S1P_2$ signaling with a specific antagonist enhances S1P-induced endothelial cell migration and angiogenic response[141].

## CARDIOVASCULAR EFFECTS OF S1P

Several studies have investigated the effects of S1P on vascular smooth muscle and vascular tone. Intravenous injection of S1P reduces renal and mesenteric blood flow[142]. Many of these studies have shown that S1P causes contraction of isolated arteries *in vitro* and vasoconstriction *in vivo*, although in some arteries S1P causes dilation, particularly in aorta when preconstricted (for reviews see [143,144,145]). For example, S1P causes contraction of renal and mesenteric[146], canine basilar[147], spiral modiolar[148], pulmonary[149], and placental[150] vessels, but dilates preconstricted aorta[149].

S1P may affect vascular tone through a number of signaling pathways. S1P stimulated contraction of cerebral artery that was mediated by ROCK downstream of Rho activation and calcium mobilization from intracellular stores downstream of PLC[151]. On the other hand, in the same study, S1P did not affect aorta and this was correlated with low levels of $S1P_2$ and $S1P_3$ expression in aorta and higher levels in cerebral artery[151]. S1P also caused contraction of coronary artery smooth muscle cells through $S1P_2$ activation[152] and increased portal pressure via Rho-mediated contraction of hepatic stellate cells[153]. Thus, it is likely that both $S1P_2$ and $S1P_3$ contribute to S1P-induced vascular smooth muscle contraction and arterial constriction through Rho and PLC pathways, respectively. Moreover, $S1P_2$ also induced increased COX-2 expression and activation, and cAMP accumulation in coronary artery smooth muscle cells, leading to prostaglandin synthesis[154]. Various mediators produced downstream of COX-2 can cause both vasoconstriction and vasodilation[155].

Dilation of preconstricted aorta was mediated by $S1P_3$, since the effect was absent in vessels from S1P knockout mice[156]. Furthermore, S1P mediates HDL-induced vasodilation via $S1P_3$ and nitric oxide production from endothelial cells in aorta[157]. In addition, $S1P_1$ has been shown to activate eNOS in endothelial cells[50] and, thus, it may also contribute to this response. Indeed, loss of $S1P_1$ expression in vessels of aged rats allows enhanced sensitivity to S1P-induced vasoconstriction[158], presumably due to $S1P_3$ signaling.

Another important role for S1P in the vasculature is endothelial barrier enhancement. This is an important step late in angiogenesis as well as a part of vascular homeostasis. S1P enhances endothelial barrier integrity through $S1P_1$ and partially $S1P_3$ signaling, leading to Rho activation and actin rearrangement[159]. Activation by S1P recruits $S1P_1$ to caveolae[50]. This step promotes activation of PI3 kinase, Tiam1-mediated Rac activation, and cortical actin rearrangement, leading to endothelial barrier enhancement[160]. Furthermore, $S1P_1$ is also transactivated by activated protein C to mediate endothelial barrier enhancement[161]. In contrast, in alveolar epithelial cells, $S1P_3$ signaling leads increased epithelial permeability by opening tight junctions[162]. The targeted deletion of $S1P_3$ prevents disruption of pulmonary epithelial tight junctions by S1P[162]. Thus, $S1P_3$ is being investigated with regard to mediating enhanced epithelial permeability observed in pulmonary edema.

S1P also regulates heart rate. Intravenously administered S1P decreases heart rate, ventricular contraction, and blood pressure in rats[163]. S1P induction of bradycardia was found to be mediated by $S1P_3$, as this effect was absent in $S1P3^{-/-}$ mice[164,165].

## ROLES OF S1P RECEPTORS IN IMMUNE CELL REGULATION

A major current focus of research on S1P, in particular through $S1P_1$ signaling, is its effects on lymphocyte trafficking. This topic has been the subject of several recent comprehensive reviews[166,167,168,169,170] and, therefore, it will be discussed only briefly here. These studies were initiated by the discovery that the immunosuppressant FTY720 is a sphingosine analog that, when phosphorylated to FTY720-P by SphK, acts as an agonist at four of the five S1P receptors, excluding $S1P_2$[38,171]. However, FTY720 causes a down-regulation of $S1P_1$ surface expression on lymphocytes, thus acting as a functional antagonist of this receptor[172,173]. This ultimately leads to sequestration of lymphocytes in lymph nodes, as an S1P gradient is responsible for chemotactically calling lymphocytes out of nodes into the lymph. FTY720-P also induces inhibition of thymic egress of T cells through down-regulation of surface $S1P_1$ expression, as $S1P_1$ is necessary to induce thymic egress of mature thymocytes[173,174]. Furthermore, $S1P_1$ signaling is also required for retention of B cells in the splenic marginal zone[175,176].

S1P at low concentrations is chemotactic for lymphocytes through $S1P_1$ signaling[104], while at high concentrations, S1P inhibits lymphocyte chemotaxis towards chemokines[177]. This may modulate lymphocyte circulation, as the high concentrations of S1P in blood repress chemokine-driven lymphocyte movement into lymph nodes. Activation by antigen leads to down-regulation of $S1P_1$ expression, allowing lymphocytes to be called into lymph nodes. Subsequent re-expression of $S1P_1$ in the lymph nodes allows the S1P gradient to call lymphocytes back into lymph[166]. In addition, cyclic modulation of $S1P_1$ expression on lymphocytes regulates their transit through secondary lymphoid organs such that low levels of $S1P_1$, when the cells enter lymph nodes, allow cells to remain there until up-regulation of $S1P_1$ expression calls T cells into the lymph or splenic B cells into the circulation[178]. $S1P_1$ function on T cells may also be regulated by post-translation modification, as tyrosine sulfation of $S1P_1$ was shown to be necessary for the chemotactic response of T cells to S1P[179]. Furthermore, lymphocytes can also regulate $S1P_1$ by modulating expression of the lectin CD69, which interacts with and inhibits $S1P_1$. Thus, interferon (IFN)-$\alpha/\beta$ up-regulates CD69 to promote lymphocyte retention in lymphoid organs[180].

S1P and FTY720 can also affect trafficking of macrophages[181], eosinophils[182], dendritic cells[183,184], and hematopoietic progenitor cells[185]. Thus, $S1P_1$ is a major regulator of immune cell trafficking. In addition, $S1P_1$ has also been shown to suppress proliferation of naïve T cell[87,88]. T cell activation leads to $S1P_1$ down-regulation, allowing proliferation[104].

T cells also express $S1P_4$ as well as $S1P_1$. While $S1P_1$ controls T cell migration, $S1P_4$ plays roles in T cell proliferation and cytokine secretion[186]. Secretion of interleuken-4 (IL-4), IL-2, and IFN-$\gamma$, cytokines with positive regulatory effects on immune responses, was decreased in T cells through $S1P_4$ signaling, while secretion of the inhibitory cytokine IL-10 was increased. Other S1P receptors modulate cytokine secretion as well. S1P induces release of IL-6 and IL-8 from immature dendritic cells primed with lipopolysaccharide, which express $S1P_1$ and $S1P_3$, although the specific receptor involved was not identified[187]. Furthermore, S1P treatment of maturing dendritic cells decreased secretion of TNF-$\alpha$ and IL-12, but increased IL-10 secretion, thus favoring a Th2 response from T cells[188]. Thus, S1P is an important regulator of the immune system, not only by affecting lymphocyte trafficking, but also by regulating cytokine secretion (reviewed in [189]).

$S1P_4$ signaling inhibits T cell proliferation[186]. On T cell activation, $S1P_4$ is down-regulated, thus allowing proliferation[104]. Furthermore, it is interesting to note that FTY720-P selectively down-regulates surface $S1P_1$ expression, while leaving $S1P_4$, for which FTY720-P functions as an agonist, available on the cell surface[172]. This may enhance the immunosuppressive activity of FTY720, which may involve a combination of $S1P_1$ internalization and, hence, lymphocyte sequestration and $S1P_4$-mediated inhibition of proliferation.

S1P plays an additional role in immune cell regulation by controlling both chemotaxis and degranulation of mast cells. IgE binding to Fc$\varepsilon$RI triggers SphK activation and autocrine signaling of S1P through $S1P_1$, which enhances chemotaxis toward antigen. In addition, $S1P_2$ expression is up-regulated by Fc$\varepsilon$RI signaling, and $S1P_2$ signaling inhibits migration and causes mast cell degranulation[190,191].

FTY720 has tremendous therapeutic potential and is currently being studied in clinical trials. FTY720 administration to cause lymphocyte sequestration through surface down-regulation of $S1P_1$ is being investigated to treat transplant rejection and multiple sclerosis[192,193,194,195]. Since $S1P_1$ influences a variety of biological activities, additional therapeutic roles for FTY720 and other compounds, with perhaps enhanced selectivity for various S1P receptors, will likely arise with applications to cancer and other immune disorders.

## S1P AND ITS RECEPTORS IN CANCER

Significant evidence has linked S1P to several aspects of malignancy. Much of this has come through studies on the enzyme that synthesizes S1P, SphK. SphK1 overexpression transforms NIH 3T3 fibroblasts, suggesting a potential oncogenic role[196]. Overexpression of SphK1 was found in several human tumor types, including breast, lung, and colon tumors, compared to matched normal tissue, suggesting that this enzyme may play a role in a wide variety of tumor types[197]. SphK1 is also overexpressed in chemically induced, rat colon adenocarcinomas[198] and a mouse leukemia model[199]. Overexpression of SphK1 in MCF-7 breast cancer cells caused enhanced proliferation, decreased apoptosis, and led to formation of larger tumors in nude mice in an estrogen-dependent manner[200]. Conversely, a dominant-negative form of SphK1 inhibited estrogen-mediated mitogenic signaling in MCF-7 cells and decreased tumor formation in nude mice[201]. In addition, SphK overexpression also prevents apoptosis induced by serum withdrawal or chemotherapeutic drugs in several cancer types[199,202,203].

As discussed above, $S1P_2$ is known to inhibit cell migration. This effect has been found in cancer cells as well. $S1P_2$ inhibited migration and invasion through Matrigel of melanoma cells by inhibiting rac activation[204]. Furthermore, S1P treatment prevented lung metastasis of $S1P_2$-expressing melanoma cells in mice, thus demonstrating an *in vivo* significance of this effect[205].

Our group has focused on the roles played by SphK and S1P in regulating the malignant behavior of human gliomas. We recently found that high levels of SphK1 expression correlate with poor survival of patients with the most malignant form of brain tumor, glioblastoma multiforme[206]. Thus, patients with low SphK1 levels survived more than three times as long as those with high levels. Moreover, SphK knock down by RNA interference in glioma cell lines decreased cell proliferation by preventing entry of cells into the cell cycle[206].

Using a panel of human glioma cell lines, we determined that S1P stimulated proliferation of 50%, and motility and invasiveness of 60% of human glioma cell lines tested[103]. S1P-induced mitogenesis was mediated in part by $G_i$ signaling as it was approximately 50% inhibited by PTX, but required ERK and PI3 kinase activation, as inhibitors of these pathways completely blocked this response[80]. On the other hand, stimulation of motility by S1P required $G_i$ signaling, as it was completely inhibited by PTX. The motility response was partially dependent on ERK and PI 3-kinase signaling. In addition, S1P enhanced glioma cell motility regardless of whether or not it was present in a concentration gradient, indicating that S1P stimulates a chemokinetic response in these cells[103]. Thus, S1P derived from the glioma cells themselves and stimulating in an autocrine fashion would be expected to enhance cell migration and invasion. In this regard, it has been shown that S1P is released extracellularly by astrocytes[207,208] and C6 glioma cells[209].

Whether a glioma cell line responds to S1P with proliferation or motility, or both or neither, is due to the profile of S1P receptor expression. Thus, although the glioma cell lines used all express the three S1P receptors, $S1P_1$, $S1P_2$, and $S1P_3$, the relative levels of expression of these receptors vary. Cell lines that do not respond mitogenically to S1P expressed extremely low levels of the receptor $S1P_1$[103], suggesting that this receptor is crucial for mediating S1P-stimulated glioma cell proliferation. In agreement, recent data indicate that $S1P_1$ overexpression in glioma cells stimulates their proliferation more potently than overexpression of $S1P_2$ or $S1P_3$, while $S1P_1$ knock down by RNA interference is most efficacious at blocking growth of glioma cells (NY and JVB, manuscript in preparation).

Conversely, glioma cells in which S1P stimulates motility express high proportions of $S1P_1$ and $S1P_3$, relative to $S1P_2$[103]. By overexpressing or knocking down S1P receptor expression in glioma cells, Lepley et al. showed that $S1P_2$ mediates inhibition of glioma migration, while $S1P_1$ mediates enhanced migration in response to S1P[110]. Our recent results confirm that $S1P_1$ or $S1P_3$ overexpression enhances glioma cell motility and invasiveness, while $S1P_2$ overexpression decreases motility. Surprisingly, however, $S1P_2$ overexpression enhances invasion of glioma cells through Matrigel, and this response appears to be mediated through an enhanced ability of $S1P_2$-overexpressing glioma cells to adhere to Matrigel (NY and JVB, manuscript in preparation).

Malchinkhuu et al. confirmed that S1P inhibits migration of some glioma cell lines through $S1P_2$ signaling[210]. They also suggested that $S1P_2$ is up-regulated in astrocytoma cells in comparison to normal astrocytes based on receptor expression in glioma cell lines and glioblastoma multiforme (GBM) tissue[210]. However, their analysis of GBM tissue utilized only two cases. We recently examined expression levels of $S1P_1$, $S1P_2$, and $S1P_3$ by real-time PCR analysis in 48 cases of GBM in comparison to 20 cases of the relatively benign pilocytic astrocytoma. We found no significant difference in expression of $S1P_1$, $S1P_2$, or $S1P_3$ between these two tumor types[206]. However, $S1P_2$ expression in GBMs was lower than that of $S1P_1$ or $S1P_3$. Thus, although its expression level is high in some long-term glioma cell lines, $S1P_2$ is not likely to be a dominant S1P receptor in gliomas *in vivo*. This suggests that the promigratory effect of S1P may be dominant in glioma cells *in vivo*.

S1P may also be very important as a stimulator of tumor angiogenesis and as a therapeutic target to block this crucial aspect of cancer behavior. FTY720, which down-regulates $S1P_1$ expression, blocked tumor angiogenesis in a melanoma mouse model and inhibited growth of metastatic tumors[211]. Furthermore, a monoclonal antibody specific for S1P was used in animal models of several different types of tumors[212]. Without exception, the antibody decreased tumor growth potently and this was shown to be due to blocking of tumor angiogenesis. Interestingly, this was true even for a tumor cell line that is not responsive to S1P in terms of proliferation, migration, or prevention of apoptosis, suggesting that angiogenesis is a critical aspect of S1P function in malignancy.

## SUMMARY

The discovery of S1P receptors has led to an explosion of information regarding the biological significance of this bioactive lipid. In a relatively short time, numerous signaling pathways regulated by these receptors, as well as their involvement in a variety of physiologic and pathologic processes particularly within the immune and cardiovascular systems as well as in neoplastic disease, have come to light. Nevertheless, the ubiquitous nature of S1P and its receptors within most cells and tissues of the body suggest that other roles played by this signaling system remain to be discovered. Knockout mouse models have provided clear functions for at least one of the receptors, $S1P_1$, as well as some information regarding the remaining receptors. However, it seems likely that functional redundancies, certainly among the S1P receptor family and possibly even with other receptors such as the LPA receptors, are masking other important functions of this system.

Given the diversity of dramatic effects described above for these GPCRs, the eventual understanding of the roles played by each subtype will likely have great medical impact. In 2001, 50% of current, commercially available pharmaceuticals targeted GPCRs and were a $30 billion industry[213]. The potential therapeutic application of S1P receptor-G-protein signaling can be applied to a variety of settings, most notably cancer and immune system disorders. Many of the aspects of malignant behavior of cancers are regulated by S1P receptor activation, including proliferation, cell survival by resistance to apoptosis, invasion, and angiogenesis. Additionally, S1P migratory effects are an integral influence on lymphocyte trafficking. Thus, the near future will undoubtedly see a continuation of the intense interest in this field.

## ACKNOWLEDGMENTS

Work in the author’s laboratory was supported by Grant # R01 NS41517 from the National Institute of Neurological Disorders and Stroke (NINDS) to JRVB.
